# Measurement of retina/choroid complex perfusion with magnetic resonance imaging in eyes with acute primary angle-closure

**DOI:** 10.5935/0004-2749.20220034

**Published:** 2025-08-21

**Authors:** Gabriel Ayub, Brunno M. Campos, Thiago J. R. Rezende, Fernando Cendes, José Paulo Cabral de Vasconcelos, Vital Paulino Costa

**Affiliations:** 1 Department de Ophthalmology, University of Campinas, Campinas, Brazil; 2 Neuroimaging Laboratory, University of Campinas, Campinas, Brazil; 3 Medical Physics Laboratory, University of Campinas, Campinas, Brazil

**Keywords:** Angle-closure glaucoma, Magnetic resonance imaging, Gadolinium, Retina, Perfusion, Glaucoma de ângulo fechado, Imagem por ressonância magnética, Gadolínio, Retina, Perfusão

## Abstract

**Purpose:**

To measure retina/choroid complex perfusion with magnetic resonance imaging in eyes with acute primary angle-closure (APAC).

**Methods:**

Three sequences of magnetic resonance imaging, two anatomical and one perfusional using gadolinium, were acquired in patients who were diagnosed with acute primary angle-closure. Regions of interest were drawn on the perfusional sequence and overlaid to the anatomical sequence. The relative blood volume measured during the first 2 s was considered as the baseline value and the change during the subsequent 28 s was analyzed.

**Results:**

Five eyes of 5 patients with acute primary angle-closure were included (3 with unilateral and 2 with bilateral acute primary angle-closure). Three contralateral eyes and 2 eyes of 2 healthy patients, paired for age and sex, were included in the control group. Acute primary angle-closure patients included 4 (80%) women, with an average age of 65.8 ± 12.37 y, mean intraocular pressure of 56.2 ± 14.67 mmHg, mean arterial pressure of 113.4 ± 8.17 mmHg, and average ocular perfusion pressure of 57.2 ± 13.46 mmHg. In the control group, the mean intraocular pressure was 15.6 ± 2.61 mmHg (p=0.0625), the mean arterial pressure was 107.4 ± 6.57 mmHg (p=1.00), and the average ocular perfusion pressure was 91.8 ± 6.72 mmHg (p=0.0625). The relative blood volume of the retina/choroid complex was -0.127 ± 0.048 in acute primary angle-closure patients and -0.213 ± 0.116 in the controls (p=0.3125).

**Conclusion:**

The magnetic resonance imaging sequence with gadolinium did not show a change in the retina/choroid complex perfusion in the eyes of patients with acute primary angle-closure.

## Abstract (pt)

**Objetivos:**

Mensurar a perfusão do complexo retina/ coróide com ressonância magnética em olhos com fechamento angular primário agudo (FAPA).

**Métodos:**

Três sequências de ressonância magnética, duas anatômicas e uma de perfusão com gadolínio, foram adquiridas em pacientes com fechamento angular primário agudo. Regiões de interesse foram desenhadas na sequência de perfusão e sobrepostas à sequência anatômica. O volume de sangue relativo nos 2 primeiros segundos foi considerado como referência, e sua variação nos 28 segundos subsequentes foi analisada.

**Resultados:**

Cinco olhos de 5 pacientes com fechamento angular primário agudo foram incluídos (3 com crise unilateral e 2 com crise bilateral). Três olhos contralaterais e 2 olhos de 2 pacientes saudáveis, pareados por sexo e idade, foram incluídos no grupo controle. Pacientes com fechamento angular primário agudo incluíam 4 (80%) mulheres, com idade média de 65,8 ± 12,37 anos, pressão intraocular média de 56,2 ± 14,67 mmHg, pressão arterial média de 113,4 ± 8,17 mmHg e pressão de perfusão ocular de 57,2 ± 13,46mmHg. No grupo controle, pressão intraocular média foi de 15,6 ± 2,61 mmHg (p=0,0625), pressão arterial média de 107,4 ± 6,57 mmHg (p=1,00) e pressão de perfusão ocular de 91,8 ± 6,72 mmHg (p=0,0625). O volume de sangue relativo do complexo retina/ coróide foi de -0,127 ± 0,048 nos olhos em fechamento angular primário agudo e -0,213 ± 0,116 nos olhos controles (p=0,3125).

**Conclusões:**

A sequência de ressonância magnética com gadolínio não demonstrou diferença na perfusão de retina/coroide em olhos com fechamento angular primário agudo.

## INTRODUCTION

Glaucoma is a progressive optic neuropathy and represents the main cause of irreversible blindness across the globe^(1)^. Primary angle-closure can be defined as the presence of iridotrabecular contact of ≥180° along with peripheral anterior synechiae and/or elevated intraocular pressure (IOP)^(2)^.

The main underlying mechanism for acute primary angle-closure (APAC) is pupillary block^(3)^, when the iris touches the lens and blocks the aqueous humor flow from the posterior chamber to the anterior chamber, dislocating the iris anteriorly and decreasing the outflow via the trabecular meshwork, resulting in increased IOP, ocular pain, conjunctival hyperemia, and blurred vision. Ocular perfusion pressure (OPP) can be defined as the difference between the average arterial pressure (MAP) and the IOP^(4)^. During an APAC attack, the IOP elevation leads to lower OPP that may lead to ischemia of the ocular vascular beds^(4,5)^. However, to our knowledge, ocular blood flow has not been measured during APAC.

The perfusion of the retina/choroid complex is given by the short posterior ciliary arteries and the central retina artery^(4)^. Several methods have been used for evaluating ocular perfusion of different vascular beds, such as color Doppler imaging (retrobulbar vessels), fluorescein angiography (retinal circulation), optic coherence tomography (OCT), angiography (optic nerve and retinal circulation) and magnetic resonance imaging (MRI)^(6-13)^. MRI is largely used in medicine and provides anatomical, functional, and perfusional images^(8,14)^, with good resolution, no need of ionizing radiation, and no examiner dependence. In the field of ophthalmology, MRI offers the advantage of not depending on clear media^(8)^. However, in comparison to other methods that measure ocular perfusion, MRI is more expensive, image acquisition takes longer, and it may become invasive if contrast is employed^(15-17)^. Gadolinium, a contrast medium that enhances the quality of images and decreases the time of acquisition^(14-16)^, is frequently used in MRI, with rare adverse effects^(17)^.

Few studies have examined the use of MRI for evaluating retina/choroid perfusion^(9-13)^. None of these studies applied a sequence that included the use of gadolinium. Furthermore, to our knowledge, no study has measured retina/choroid perfusion during an APAC attack with any method. The present study aimed to examine retina/choroid complex perfusion with MRI in the eyes of patients with APAC.

## METHODS

This cross-sectional study was approved by the Ethics Committee of the University of Campinas and was performed as per the principles in the Helsinki Declaration. All the procedures were fully explained to all the study subjects and informed consent was obtained from all the participants.

### Inclusion and exclusion criteria

We included patients with APAC, defined as those with ≥180° of iridotrabecular contact on gonioscopy, pupillary block (mydriasis, shallow anterior chamber and iris bombé), conjunctival hyperemia, corneal edema, and IOP >40 mmHg^2^. Most patients reported symptoms, such as ocular pain, blurred vision, nausea, and headache. If the patient had bilateral APAC, the eye with the highest IOP was included. As per the exclusion criteria, those with previous intraocular surgery; secondary angle-closure; ocular trauma; and other conditions, such as age-related macular degeneration, retinal detachment, panretinal photocoagulation, and amblyopia were excluded. Owing to the use of intravenous contrast, we excluded patients with a glomerular filtration rate (GFR) of <30 mL/min/1.73 m^2^ (calculated as per the Modification of Diet in Renal Diseases Study equation^(18)^), moderate or severe hepatic disease (based on the Child-Pugh score^(19)^), and a previous reaction to gadolinium. Furthermore, patients with metallic implants were excluded due to the MRI restrictions. In the control group, we included the fellow eye of patients with unilateral APAC. If it was not possible to use the fellow eye, we included healthy individuals (with open angles on gonioscopy, IOP <20 mmHg, and optic nerves with no evidence of glaucomatous damage), paired for sex and age, as controls. The exclusion criteria for the control group were the same as those for the APAC group.

The Choyke questionnaire that comprised 6 questions to assess the presence of previous renal disease or surgery, high blood pressure, diabetes and gout, was administered to all the study subjects^(20)^. If the questionnaire indicated the presence of any of these conditions, blood samples were collected to determine the GFR.

### MRI technique

A 3-Tesla MRI (Achieva, Philips Medical Systems, Best, The Netherlands) and a 32 head coil (dStream Head 32ch coil, Philips Medical Systems, Best, The Netherland) were used to obtain the images. The first sequence was an axial T1 3D, with resolution of acquisition 0.89 × 0.9 × 0.9 and reconstruction 0.45 × 0.45 × 0.45, field of vision 150 × 150 × 40, flip angle 8, time of echo 2.5 ms, and time of repeat 5.6 ms (time of sequence: 5 min and 19 s). The second sequence had the same parameters as the first, with different time of echo (4.3 ms) and time of repeat (9 ms), in addiction to fat suppression (time of sequence: 5 min and 20 s). The third sequence was a spin echoecho-planar imaging (SE-EPI), with fat suppression, field of vision 120 × 120 × 35, voxel 1.88 × 2.5, 5-mm slice thickness, 30 dynamic scans, time of echo 50 ms, time of repetition 1000 ms, and flip angle 75°. Gadolinium infusion was started at the same time as the perfusion sequence (time of sequence: 33 s). The average total time for the examination was 11 min and 12 s.

### Treatment of APAC

**Figure 1:**
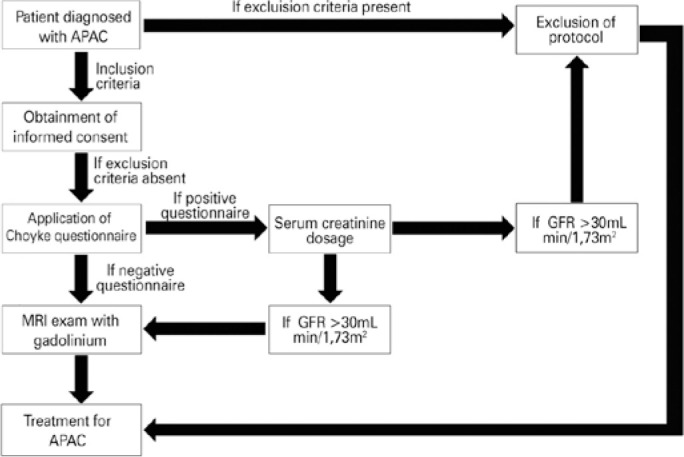
Recruitment of patients for the protocol.

After the acquisition of the MRI sequence, blood pressure was measured in the orthostatic position. The patients were treated for APAC with topical pilocarpine 2%, timolol 0.5%, brimonidine 1%, prednisolone 1%, brinzolamide 1%, oral acetazolamide 250 mg, and intravenous mannitol 20% 250 mL. Laser peripheral iridotomy was performed in both the eyes as soon as the cornea became transparent (Figure 1). The interval between diagnosis and treatment initiation ranged from 45 min to 60 min.

### Analysis of the sequences

**Figure 2:**
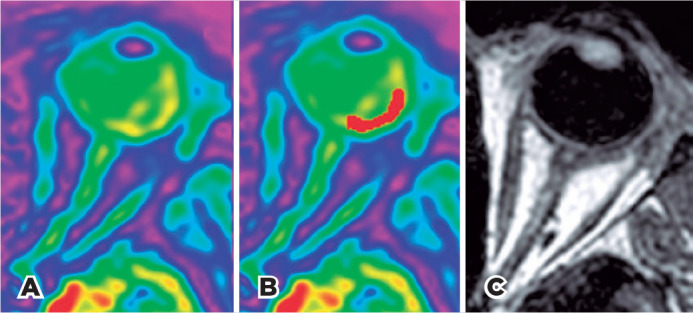
(A) perfusion sequence used as reference; (B) ROI drawn in red; (C) anatomic sequence which the perfusional sequence and the ROI were overlaid for alignment.

The dynamic sequence (EPI or perfusion/Dynamic Susceptibility Contrast (DSC)) was realigned to correct for head motion during acquisition and registered to the anatomical T1-WI with the SPM12 toolbox (Statistical Parametric Mapping 12, https://www.fil.ion.ucl.ac.uk/spm/) (Figure 2C). In order to measure retina/choroid perfusion, a region of interest (ROI) was drawn in the sequence (Figure 2A) limited by the edge of the optic disc and the insertion of the lateral rectus muscle (Figure 2B) using the software MRIcron (http://mricron.com). In order to calculate the perfusion as the relative blood volume (rBV), the mean value of the first 2 s of the perfusional sequence was considered as baseline, and the rBV in the subsequent 28 s was measured relative to the baseline value. The values were calculated with a personalized script Cr(t) = - (1/te) ln(S(t)/S(0)) using the Matlab 2017 (The Math Works Inc., Natick, MA, USA), where “Cr” stands for relative concentration; “t”, instant; “te”, time of echo; “S(t)”, signal on that instant; and “S(0)”, the signal of reference.

Mean ocular perfusion pressure (MAP - IOP) was calculated using the IOP measured at baseline. MAP was defined as MAP=(2 × diastolic pressure + systolic pressure)/3.

### Statistical analyses

Statistical analyses were performed using the SAS System for Windows version 9.4 (SAS Institute Inc., 2002-2008, Cary, NC, USA) and R (R Foundation for Statistical Computing, Vienna, Austria). Graphics and tables were created with Microsoft Office Excel 2016 (Microsoft Corporation, Redmond, WA, USA). The results are expressed as mean ± standard deviation values. The Wilcoxon test was applied to compare the IOP, MAP, OPP, and rBV between the groups. The Spearman correlation coefficient was used to evaluate the correlation between rBV-IOP and rBV-OPP. A p-value £0.05 was considered to indicate statistical significance.

## RESULTS

From September 2018 to September 2019, 15 patients were diagnosed with APAC. Of these, 3 (20%) had bilateral APAC, 11 (73.33%) were women; the average participant age was 63.4 ± 8.66 y, and the mean IOP in the APAC eyes was 50.44 ± 13.38 mmHg. Ten eyes were excluded from the study; 6 arrived at an emergency room when the MRI machine was unavailable, 2 had metallic implants, 1 had hepatic insufficiency, and 1 refused to participate in the study.

Thus, 5 patients were enrolled in the study; 4 (80%) of these were women, with a mean age of 65.8 ± 12.37 y. Three patients had unilateral APAC, and 2 had bilateral APAC. The mean IOP of the APAC eyes was 56.2 ± 14.67 mmHg, the average MAP was 113.4 ± 8.17 mmHg, and the mean OPP was 57.2 ± 13.46 mmHg.

The control group included 3 fellow eyes of patients with unilateral APAC and 2 eyes of 2 healthy patients, paired for sex and age. The average IOP of the control eyes was 15.6 ± 2.61 mmHg (p=0.0625), the mean MAP was 107.4 ± 6.57 mmHg (p=1.00), and the mean OPP was 91.8 ± 6.72 mmHg (p=0.0625) (Tables 1 and 2).

**Table 1:** Laterality, age, IOP, MAP, OPP, and rBV of the enrolled patients with APAC and the controls. Patients 3, 6, and 7 correspond to the ones that had unilateral APAC. The eyes of patients 2 and 5 correspond to healthy individuals paired for sex and age and included as controls. Age, expressed in years; IOP; MAP; and OPP expressed in mmHg

Patient	Group	Laterality	Age	IOP	MAP	OPP	rBV
1	APAC 1	OS	67	64	127	63	-0.202
2	Control 1	OD	65	14	97	83	-0.155
3	APAC 2	OD	73	47	107	60	-0.122
3	Control 2	OS	73	16	107	91	-0.189
4	APAC 3	OD	49	78	113	35	-0.130
5	Control 3	OS	51	18	113	95	-0.312
6	APAC 4	OS	81	42	113	71	-0.071
6	Control 4	OD	81	12	113	101	-0.062
7	APAC 5	OD	59	50	107	57	-0.110
7	Control 5	OS	59	18	107	89	-0.345

APAC= acute primary angle-closure; OD= right eye; OS= left eye; IOP= intraocular pressure; MAP= mean arterial pressure; OPP= ocular perfusion pressure; rBV= relative blood volume.

**Table 2:** Comparison of IOP, MAP, OPP, rBV, and p-value on the APAC group and control group. Values are expressed as mean ± standard-deviation values. The p-value was not significant in the analysis because of the low number of enrolled patients. IOP, MAP, and OPP expressed in mmHg

	APAC	Control	p-value
**IOP**	56.2 ± 14.67	15.6 ± 2.61	0.0625
**MAP**	113.4 ± 8.17	107.4 ± 6,57	1.00
**OPP**	57.2 ± 13.46	91.8 ± 6.72	0.0625
**rBV**	-0.127 ± 0.048	-0.213 ± 0.116	0.3125

APAC= acute primary angle-closure; IOP= intraocular pressure; MAP= mean arterial pressure; OPP= ocular perfusion pressure; rBV= relative blood volume.

Wilcoxon test was used to calculate the p-value.

**Figure 3:**
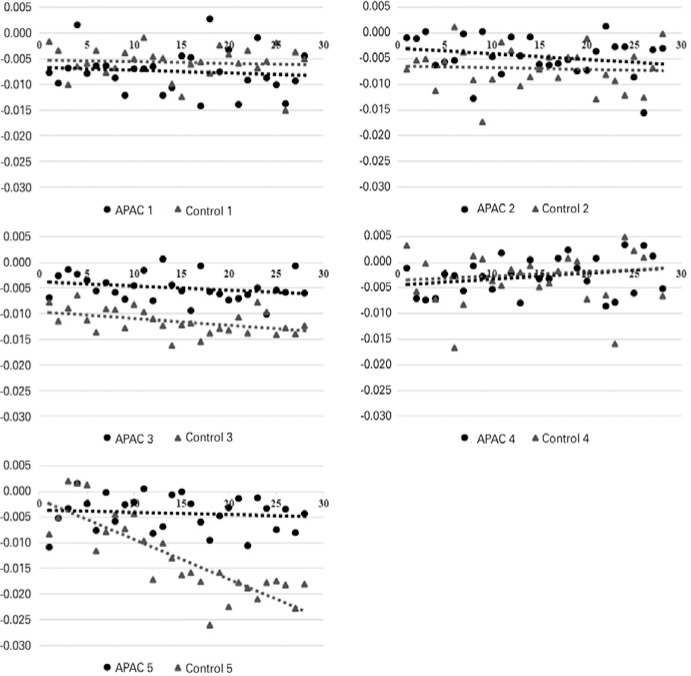
rBVs during the 28 s in APAC eyes (black dots) and controls (grey dots). The tendency line of each patient is shown. Perfusion was calculated by the area limited by the tendency line and the horizontal axis 0. The vertical axis represents the signal intensity (arbitrary units) and the horizontal axis represents the time (in seconds). In patients 3, 6, and 7 (Control/APAC 2, 4, and 5, respectively), the contra-lateral eye was used as the control. Patient 1 (APAC 1) was paired with patient 2 (Control 1), and patient 4 (APAC 3) was paired with patient 5 (Control 3).

**Figure 4:**
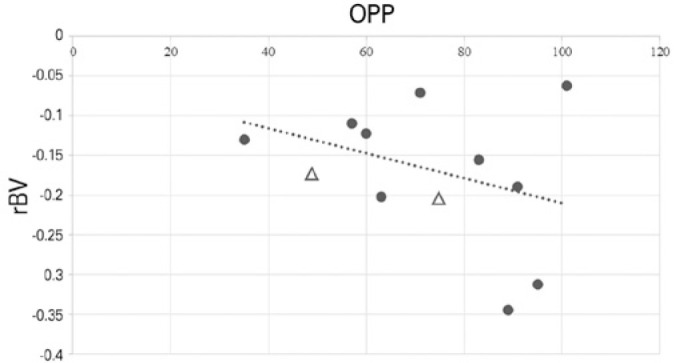
Correlation between OPP, expressed in mmHg, and rBV (r=-0.18, p=0.6073). Circles represents the eyes included on the analysis. Triangles represent the contra-lateral eyes of patients with bilateral APAC who were not included in the statistical analyses.

**Figure 5:**
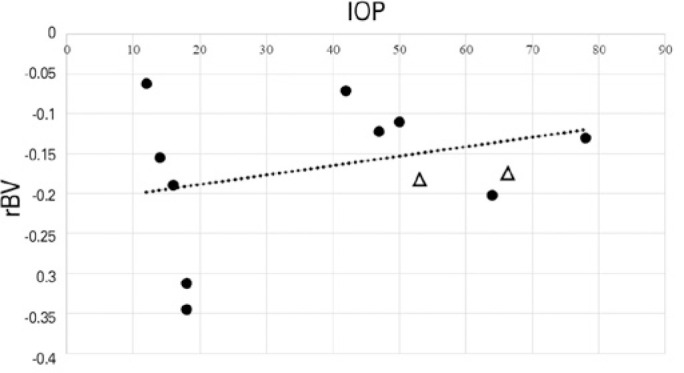
Correlation between IOP, expressed in mmHg, and rBV (r=0.036, p=0.9319). Circles represent the eyes included in the analyses. Triangles represent the contra-lateral eyes of patients with bilateral APAC who were not included in the statistical analyses.

The average rBVs of the retina/choroid complex were -0.127 ± 0.048 and -0.213 ± 0.116 (p=0.3125) in the APAC and control groups, respectively (Table 2 and Figure 3). There was a weak negative correlation between rBV and OPP (r=-0.18; p=0.6073) (Figure 4) and a weak positive correlation between rBV and IOP (r=0.036; p=0.9319) (Figure 5).

No adverse effects were reported during the MRI examination.

## DISCUSSION

We measured retina/choroid perfusion using an MRI technique with gadolinium contrast medium in normal and APAC eyes. Although the OPP was higher in the controls, the MRI protocol we used could not enable the detection of differences in the retina/choroid perfusion of the 2 groups. Figure 4 demonstrates a negative weak correlation between rBV and OPP, indicating that an increase in OPP was related to a slight decrease in rBV. figure 5 shows a weak positive correlation between IOP and rBV, suggesting that an increase in IOP was related to a slight increase in rBV. MRI studies that measure the perfusion with rBV and use DSC techniques showed that the perfusion is measured indirectly by the drop of the sign caused by the gadolinium, which will be discussed next. The values are always expressed in negative values, with a more negative value indicating a higher perfusion in the area, and a less negative value reflecting lower perfusion^(21)^. A decrease in rBV, relative to an increase on OPP, should be interpreted as indicative of better perfusion, while an increase in rBV, relative to an increase on IOP, indicates worse perfusion. However, the correlations between rBV-OPP and rBV-IOP were weak and non-significant, potentially owing to the relatively small sample size of our study or to a limitation in the technique that we used to measure the choroid/ retina perfusion. In fact, the statistical power of finding a significant difference between APAC eyes and controls with our small sample size (n=5) was 37.88%. For a statistical power of 80%, the sample size would have to be increased to 11 in each group. Other studies that used MRI also found weak correlations between ocular blood flow and IOP or OPP. Zhang et al.^(12)^ found a weak, non-significant positive correlation between OPP and ocular blood flow in the choroid (p>0.05), except in one of the 4 included subjects^(12)^. Nateras et al.^(11)^ found weak positive correlations between choroidal blood flow and OPP (r=0.13, p=0.6) and MAP (r=0.14, p=0.6), while a weak negative correlation was found between choroidal blood flow and IOP (r=-0.15, p=0.9). As per these studies, we found no significant correlation between ocular perfusion and OPP or IOP. Previous studies also had a smaller sample; this reduced their statistical power. Thus, we believe that the relatively small sample size is a possible explanation for the absence of a correlation between choroidal/retinal perfusion and OPP/ IOP. The other explanation is related to the method. The technique we described may not be accurate enough to allow the detection of a correlation between these parameters.

Previous studies measured retina/choroid perfusion on mice^(22)^ and humans^(9-13)^ using MRI techniques ([arterial spin label (ASL)] without using gadolinium. Instead of contrast, ASL-MRI uses the blood water protons as endogenous markers for quantifying perfusion^(23,24)^. The technique involves the acquisition of two sequences (label image and control image), and perfusion is extracted from the signal difference between these two sequences^(23,24)^.

The first study to discuss the use of MRI for measuring retina/choroid perfusion was published by Maleki et al.^(9)^, who evaluated 5 healthy individuals using a 3-Tesla MRI machine and an 8-channel commercial coil, and obtained a mean value of 261 ± 87 mL/100 mL/min^(9)^. Peng et al.^(10)^ recruited 5 healthy individuals to evaluate retinal blood flow changes induced by hypercapnia with a 3-Tesla MRI machine and a custom eye coil. They reported a 12% ± 4% (from 93 ± 31 mL/100 mL/min to 104 ± 35 mL/100 mL/min, p<0.01) increase in the retinal blood flow induced by hypercapnia. Nateras et al.^(11)^ evaluated the foveal and optic nerve perfusion and its correlation with age in 17 healthy subjects. The foveal perfusion was 295 mL/100 mL/min, with an annual reduction of 2.7 mL/100 mL/min (r=-0.7, p=0.003); the optic nerve head perfusion was 112 mL/100 mL/ min, with a decrease of 0.94 mL/100 mL/min per year (r=-0.5, p=0.05). No significant correlation was found between blood flow and OPP (r=0.13, p=0.6), MAP (r=0.14, p=0.6), or IOP (r=-0.15, p=0.9). Zhang et al.^(12)^ used a 3-Tesla MRI machine to measure retina/ choroid perfusion in 4 healthy individuals, using a 32-channel custom coil, during rest and after isometric exercise. The authors reported a baseline measurement of 149 ± 48 mL/100 mL/min that increased bey 25% ± 7% after exercise. Ocular blood flow increased in parallel with heart frequency (19% ± 8%), MAP (22% ± 5%), and OPP (25% ± 6%).

The only study to test the reproducibility of MRI measurements of the retina/choroid perfusion reported an average baseline value of 77.86 ± 29.8 mL/100 mL/ min^(13)^, lower than that reported in the previous studies. This study used a 3-Tesla MRI machine and a 32-channel coil and did not use gadolinium. Twenty healthy adults had their perfusion measured twice during the same session (intra-session), and the sequence was repeated 2 d thereafter at the same time of the first session (inter-session). They found high intra-session (ICC = 0.969; CoV = 9.3%) and intersession reproducibility (ICC = 0.885; CoV = 17.3%)^(13)^.

Our findings should be compared with previous reports carefully because there are differences in the MRI machines, coils, reconstruction, and image processing in the different studies, and these factors influence the results and their interpretation. Moreover, several limitations have been reported with the use of ASL MRI for assessing retina/choroid perfusion. This technique cannot evaluate the perfusion of the retina and choroid separately owing to low spatial resolution, eye motion, and/ or low signal strength of the applied techniques^(9,11,12)^. In addition, authors reported several issues in measuring eye perfusion. Peng et al.^(10)^ had issues on calculation of the perfusion, using estimated values for it, given that parameters of his sequence for eye perfusion were not previously validated. Khanal et al.^(13)^ reported challenges in quantifying the perfusion based on a ROI drawn on an anatomical image with higher spatial resolution instead of a perfusion sequence that might have underestimated the value, and used previous cerebral perfusion studies as reference to measure the retina/choroid perfusion, in the absence of valid parameters for the eye.

Perfusion is determined by subtracting one image from another, one main issue on ASL is motion, which may compromises alignment of the images^(23-25)^. Previous studies minimized motion using a fixation target and/ or blink and breathing synchronization^(11)^. Furthermore, ASL protocols usually involve a lengthy acquisition time and low resolution. Better accuracy of perfusion measurements is expected with the use of gadolinium because it involves a shorter acquisition time, enhances the signal, and is a better marker for perfusion than non-gadolinium techniques^(14,21)^.

### Behavior of gadolinium on DSC sequences

**Figure 6:**
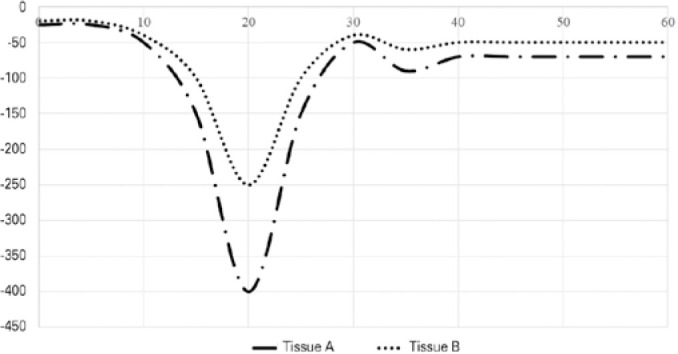
Gamma-fitting function on a MRI perfusion study using DSC technique. Tissue A had a more significant decay of the signal compared to Tissue B, indicating higher perfusion of Tissue A. The vertical axis represents the signal intensity (arbitrary units) and the horizontal axis represents the time (in seconds).

Gadolinium changes the time of relaxation and, consequently, the capture of the signal by the tissues^(26)^. When the sequence acquisition is initiated, the signal finds in a baseline, constant value. As the gadolinium starts to diffuse in the tissue via the micro vessels, on T2 and T2^*^ acquisitions, a decay on the signal is observed^(14,26,27)^. When the concentration reaches its peak, the peak of the decay is observed. Subsequently, the gadolinium is cleared from the tissue, and the signal recovers to a new baseline level^(26)^. During the recirculation of the contrast, a new decay is observed, lower than the first one, and the signal returns to a new baseline value again^(26)^. This phenomenon is well described in brain tissue and is called gamma-fitting function^(26)^ (Figure 6).

The parameters that may be analyzed on DSC^(28)^ acquisitions are arrival time (AT), the time between the start of the contrast injection and the beginning of the signal decay; time to peak (TTP), the time between the start of the contrast injection and its maximum concentration on the tissue (maximum decay); mean enhancement time (MET), the time that the contrast takes to be cleared from the tissue since the first arrival, indicated by the interval between the beginning of the decay of the signal and its recovery to baseline; and the mean transit time (MTT), the time that a single molecule of gadolinium takes to be cleared from the tissue. The perfusion can be calculated by the negative enhancement integral (NEI), defines as the area under the curve of the gamma-fitting function. In this series, the gamma-fitting function was not observed; thus, we could not calculate the AT, TTP, MET, MTT, and NEI. Thus, the retina/choroid perfusion was calculated indirectly, using the area under the tendency line that expresses the variation of gadolinium concentration on the ROI (Figure 3).

In this study, gadolinium showed a different behavior than that described in the tumor and brain tissues^(14)^. The impossibility of obtaining a gamma fitting function can be explained with several reasons. The perfusion of the retina/choroid may be limited by its relatively small size, which makes the manually drawn ROI englobe a small number of voxels, the MRI image unit^(29)^. The region is composed by different densities, such as sclera, vitreous, bones, air, and retrobulbar fat, that may interfere with the signal^(29)^. Furthermore, the region is superficial; this may increase the number of artifacts. Involuntary ocular movement, a possible source of variability, was reduced with the use of SE-EPI technique, minimizing motion^(25,30)^. Finally, to our knowledge, no studies have investigated the dynamics of gadolinium in the retina/choroid; therefore, it is difficult to determine if the gadolinium behavior we observed is real or a consequence of limitations resulting from the use of the MRI machine or technique, including non-uniform magnetic field, diminished time of echo and time of repetition, and thickness of the slice^(29)^.

### Limitations

This study has certain limitations. Apart from the unexpected behavior of gadolinium and the technical limitations already mentioned, we were unable to measure the perfusion of retina and choroid separately owing to the limited resolution of the technique. Moreover, the reproducibility was not verified because repeated administrations of gadolinium might raise the risk of adverse effects^(17)^. The heterogeneity of the control group, with the use of fellow eyes of APAC patients and healthy eyes, constitutes a selection bias, because the later may present different systemic conditions, including the use of medications that may influence ocular perfusion. Finally, the relatively small sample size is an important study limitation because it reduces the statistical power of the analyses.

In conclusion, the MRI sequence with gadolinium did not show a change in the retina/choroid complex perfusion in APAC eyes. We believe that more studies are required to improve the technique that would allow the use of DSC MRI with gadolinium to measure retina/ choroid perfusion.
